# A new diagnostic vestibular evoked response

**DOI:** 10.1186/s40463-015-0065-7

**Published:** 2015-04-11

**Authors:** Zeinab A Dastgheib, Brian Lithgow, Brian Blakley, Zahra Moussavi

**Affiliations:** Department of Electrical & Computer Engineering, University of Manitoba, Room E3-512 Eng. Bldg., 75A Chancellor’s Circle, Winnipeg, MB R3T 5V6 Canada; EVestG Research Lab, Riverview Health Centre, Room PE446, 1 Morley Avenue, Winnipeg, MB R3L2P4 Canada; Department of Otolaryngology - Head and Neck Surgery, University of Manitoba, GB421 - 820 Sherbrook Street, Winnipeg, Manitoba R3A 1R9 Canada; Department of Electrical & Computer Engineering, University of Manitoba, 75A Chancellor’s Circle, Winnipeg, MB R3T 5V6 Canada

**Keywords:** Meniere’s disease, EVestG, Vestibular response, Classification, Fractal dimension

## Abstract

**Objective:**

To describe the development of a new clinically applicable method for assessing vestibular function in humans with particular application in Meniere’s disease.

**Study design:**

Sophisticated signal-processing techniques were applied to data from human subject undergoing tilts stimulating the otolith organs and semicircular canals. The most sensitive representatives of vestibular function were extracted as “features”.

**Methods:**

After careful consideration of expected response features, Electrovestibulography, a modified electrocochleography, recordings were performed on fourteen Meniere’s patients and sixteen healthy controls undergoing controlled tilts. The data were subjected to multiple signal processing techniques to determine which “features” were most predictive of vestibular responses.

**Results:**

Linear discriminant analysis and fractal dimension may allow data from a single tilt to be used to adequately characterize the vestibular system.

**Conclusion:**

Objective, physiologic assessment of vestibular function may become realistic with application of modern signal processing techniques.

**Electronic supplementary material:**

The online version of this article (doi:10.1186/s40463-015-0065-7) contains supplementary material, which is available to authorized users.

## Introduction

Vestibular disorders are among the most common reasons that patients seek the advice of a physician, yet the diagnosis of dizziness largely relies on the patient history. The patient history is subjective and its reproducibility has not been validated. Significant physiologic disruptions of neurological function should cause repeatable, measureable changes in neural activity. We believe that sophisticated and objective measurement of these changes should be diagnostic and should reveal underlying pathologic mechanisms. This paper outlines the application of advanced statistical signal processing techniques from the fields of engineering and statistics to understand normal and pathologic vestibular function using Meniere’s disease as a prototype.

Evoked potentials have been successfully applied to diagnose auditory disorders but may be difficult for vestibular diagnosis. Auditory evoked potentials typically involve temporal averaging of several hundred auditory stimuli which may be problematic in vestibular stimuli. On the other hand, when observing the averaging process of auditory evoked potentials in real time, the first response or two are often adequate to see the general nature of the response. It would seem then that the first response or two should contain diagnostic information if it could be extracted. With this observation in mind is seems plausible that sophisticated signal processing techniques might be able tease out enough information from a few tilts to permit recognition of repeatable patterns of waveforms that could be diagnostically useful.

**Figure 1:**
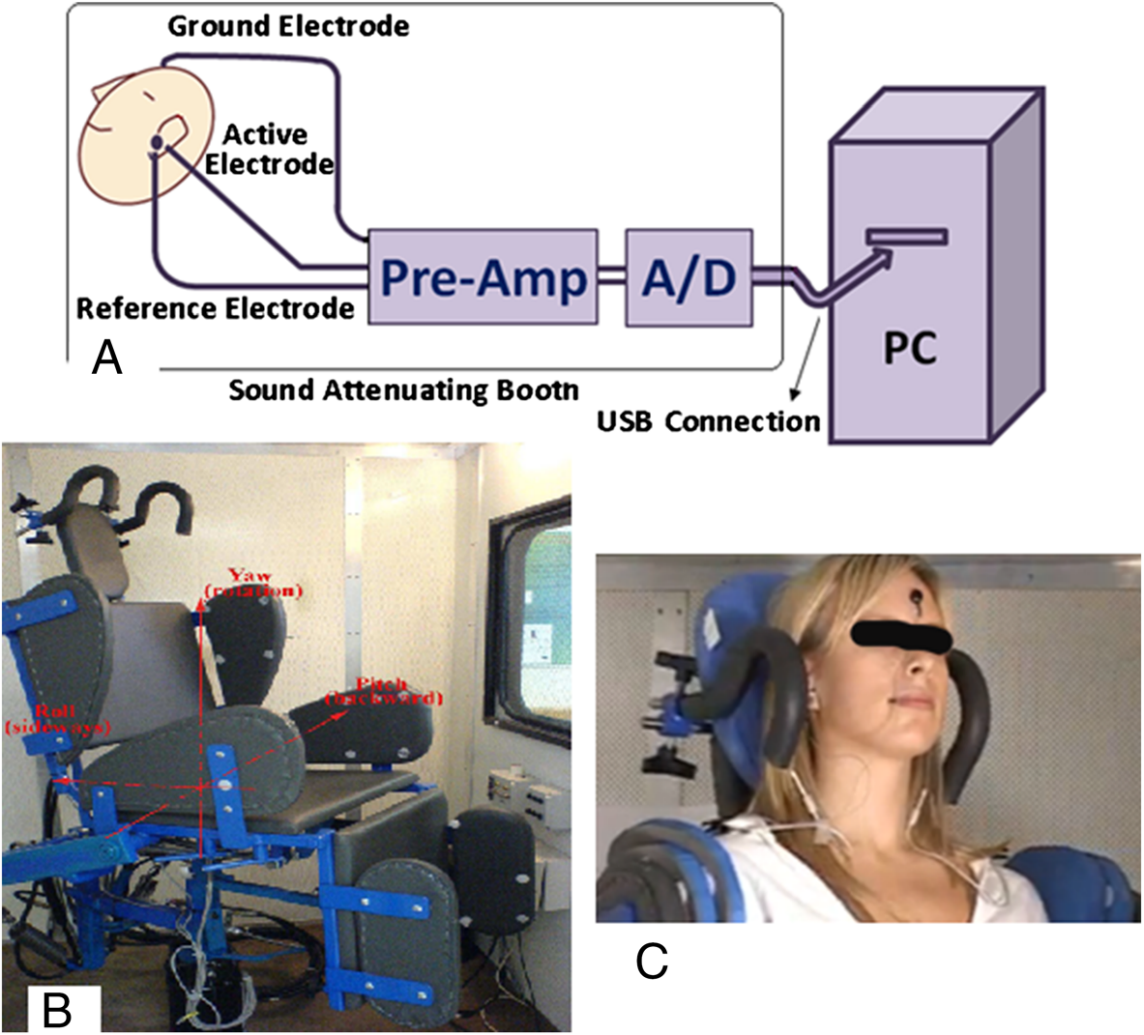
**The recording system with Hydraulic chair.** System diagram is shown in **A**. **B** displays the hydraulic chair with the axes of rotation and **C** illustrates the method of holding the subject’s head for testing while the electrodes are connected.

Electrocochleography (ECoG) is a diagnostic evoked-potential method that records an excitatory ‘gross’ evoked response by averaging responses to a series of auditory clicks [1-3]. A useful, analagous vestibular test would directly measure the dynamic response of the vestibular system to both excitatory and inhibitory inputs, and derive a measure of its dynamic range. Electrovestibulography (EVestG) [4,5] is similar to ECoG but the multiple acoustic stimuli are replaced by one or two passive whole body tilts in a hydraulically controlled chair located in an electrically and acoustically shielded chamber. The EVestG signal is recorded during dynamic and static phases via ECoG electrodes resting near the tympanic membrane of both ears [6]. Figure 1, shows the recording system with the hydraulic chair. A proprietary software algorithm called the “Neural Event Extraction Routine (NEER)” [5] has been developed to extract the field potential (FP) signals from the EVestG recordings. NEER algorithm derives two signals from the recording raw signals: the averaged response of FPs and the time intervals between the FPs. Pattern recognition techniques applied to EVestG signals have shown very encouraging results in other neurological diagnostic applications such as Parkinson’s disease, depression, and schizophrenia disorder by other studies [7-9]. In this paper will apply EvestG techniques to Meniere’s disease patients with a view to developing an objective test for the disorder.

Usually several features as biomarkers are extracted from the output of the NEER algorithm on the EVestG signals. Most diagnostic tests measure the signals’ most important parameters to classify a system as normal or abnormal. The “feature” extraction technique utilizes many quantitative criteria from the signal to categorize the response. Extracted criteria may be statistical parameters, calculations of some characteristic of the waveform or derivations from multiple other sources. The technique of “feature extraction” is similar to that used in cochlear implants. Herein, we apply it to vestibular function. A major difficutly in measuring biological electrical potentials is the signal-to-noise ratio. We are trying to detect a small signal in the midst of tremendous electrical noise from nerve, muscle and other cells. In this paper we discuss the clinical utility of NEER algorithm and EVestG extracted signals. First we briefly describe of the key concepts. Further details can be found in the Additional file 1.

### Features

In signal processing, features are quantities that are associated with a signal or a process. Features may be statistical measures such as the mean, standard deviation, skewness, kurtosis, etc. of a statistical process, or they may be other quantitative measures representing fractal nature, power distribution, etc. of a signal or process. In addition to statistical features, this report includes features representing fractal dimension (FD) as assessed by the Higuchi fractal dimension (HFD), and entropy-based dimensions such as the Information dimension (DI) [10] and the Correlation dimension (DC). These features were extracted from the FP and timing intervals of the EVestG recordings from patients undergoing EVestG testing.

**Fractal dimension calculation** (FD) can be interpreted as the “degree of meandering” (roughness, brokenness, irregularity or singularity) of an object. Another interpretation of a fractal dimension is that it is the critical exponent in a power-law relation [10,11]. Fractal dimension mathematically refers to a non-integer or fractional dimension of a self-similar (or a self-affine) object [10]. The self-similarity (or self-affinity) of the object is confirmed if a portion of the object is exactly (or statistically) a scaled down version of itself.

FD analysis is widely used as an analytical tool in a variety of research areas particularly biological signal processing [11]. It measures the irregularity, the complexity and the self-similarity of a signal. The more complex the signal, the higher the FD value will be. Two effective methods for FD calculation are the Higuchi fractal dimension and Entropy based fractal dimension (see Additional file 1 for details).

**Higuchi Fractal Dimension (HFD)** is well suited for studying signal fluctuation in one dimension [12]. HFD, proposed in 1988, is an efficient algorithm for measuring the FD of discrete time series [13]. HFD has been established as a method to characterize the morphological complexity of biological signals [14].

**Entropy-Based Fractal Dimensions -** Entropy can be defined as the amount of information needed to specify the state of a system [10]. Entropy is known as the measure of disorder in physical systems, or an amount of information that may be gained by observations of disordered systems [15]. Entropy-based fractal dimensions can deal with fractals objects which have non-uniform distributions, while the morphological fractal dimensions such as HFD deal with the shape of a projection of the fractal only. This is understandable because the morphological dimensions are purely metric and not probabilistic concepts. The information dimension (DI) and correlation dimension (DC) are special cases related to generalized entropy concept as introduced by Alfred Renyi in 1955 [16]. Both dimensions are improvements of the geometric definition of a fractal object (See Additional file 1).

The DI reveals the expected spread in the non-uniform probability distribution of the fractal objects, but not its correlation. The DC was introduced to address this problem. Both DI and DC represent a weighted average measure of the actual distribution of self-information over the fractal object (See Additional file 1).

**Linear Discriminant Analysis (LDA)** is a mathematical technique that utilizes features to classify objects or signals into one or more classifications. Each object/signal has certain features that may be relevant in classifying that object/signal; some of these features can be more important predictors than others. In this study, we are trying to classify patients as with either Meniere’s disease or no Meniere’s disease.

**Minimal-redundancy-maximal-relevance (mRMR)** feature selection method [17] is a method of ranking features based on the two criteria of minimum redundancy and maximum relevancy; thus allowing to choose the most relevant and least redundant features as the best set of features for classification.

## Methods

EVestG research labs have been established for human testing at Alfred Hospital in Melbourne Australia and Riverview Health Center in Winnipeg, Canada. In this study, however, only data recorded at Alfred Hospital in Australia has been used. The EVestG signal acquisition apparatus is illustrated in Figure 1.

### Study subjects

EVestG data of 14 Meniere’s patients (54.2 ± 9.7 years, 4 males) and 16 healthy individuals (56.1 ± 5.5 years, 7 males) from the EVestG lab at the Alfred Hospital, Melbourne, Australia, were used as the training data to design the diagnostic algorithm. Ethics approval was granted by the Health Research Ethics Board of Alfred Hospital, and all study subjects signed an informed consent form prior to the experiments.

### EVestG protocol

A complete EVestG recording [4,5] includes passive lateral whole body tilts, up/down movements, rotations from the sitting position and up/down movements and rotations from the supine position. This paper will report data for right and left lateral tilts only. Tilts were symmetric movements moving over 3 seconds from the upright sitting position to a position 45 degrees from the vertical to the right, then upright and then to the left. The EVestG signal was recorded at a sampling rate of 41666 Hz.

**Figure 2:**
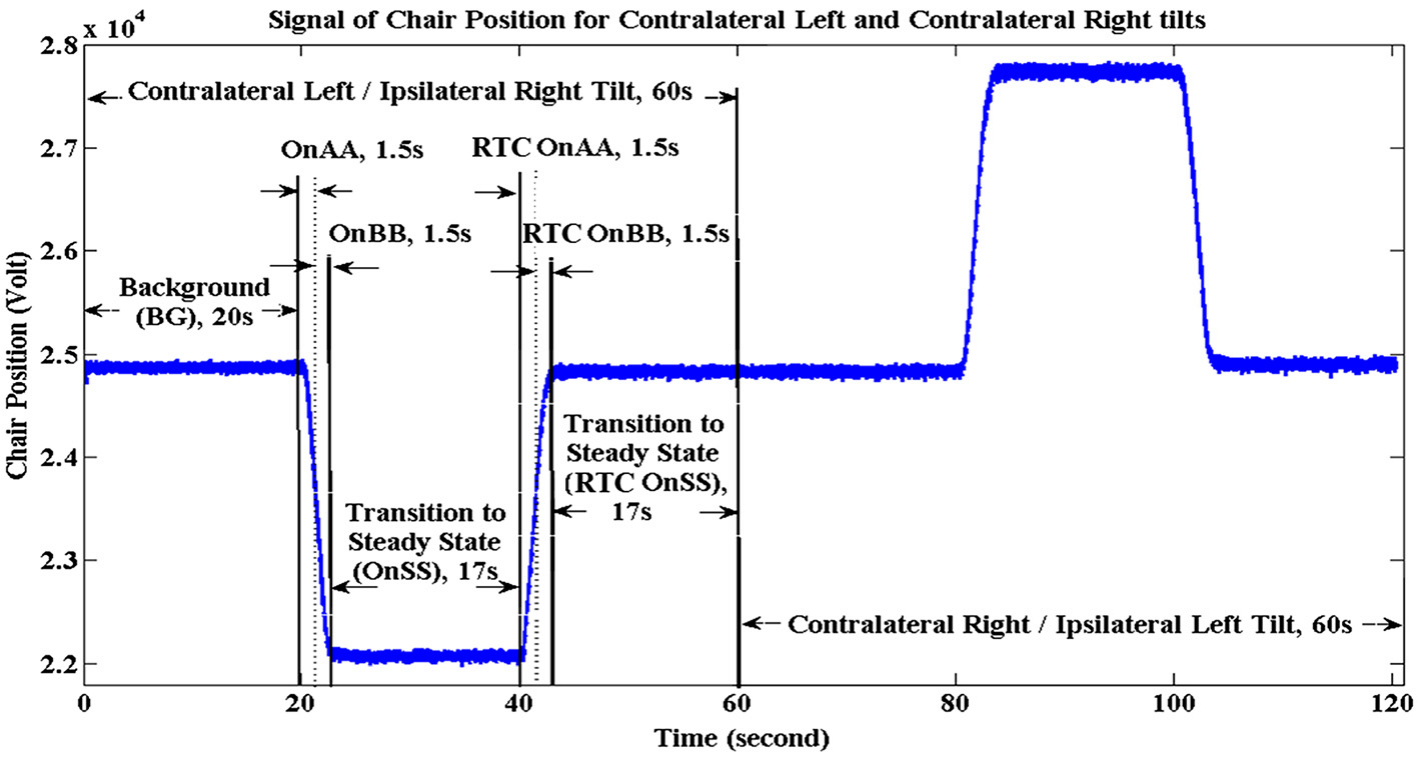
**The chair movement pattern during the side tilt.**

Table 1 shows the timing segments and names for a tilt to the right and back to the upright position. The labels for the segments in Table 1 are those from the original EvestG description in the literature that relate to EvestG in general, rather than specific application to the ear. Following this tilt to the right, a tilt to the left is performed with the same naming system. Rightward tilts are referred to as right ipsilateral (IP) and left contralateral (CT). In different segments of the motion, there are “periods of interest” that are the critical time periods for analysis as indicated in Figure 2.

**Table 1:** **Labeling of components of EvestG test**

**Segment**	**Period of Interest (POI) of the segment (See Figure** **2** **)**	**Name of POI**
20 s background recording	the final 1.5 s	BGi
3 s lateral tilt to the right (about 40 degrees)	first half, 1.5 s, the acceleration phase	On AA
	second half, 1.5 s the deceleration phase	On BB
17 s rest in the tilted position	final 1.5 s just before returning to center	RTC BGi
3 s returning back to center	the first 1.5 s	RTC OnAA
	the second 1.5 s	RTC OnBB
17 s rest at the center position before a new tilt	Transition to Steady State	RTC OnSS

Labeling of components of EvestG test.

The NEER [5] algorithm extracts data from the neural response. Each tilt’s recorded data results in two main signals, an average field potential (FP) and its firing pattern for each time segment (see Table 1) for each ear. In this study, we only used signals of the contralateral and ipsilateral side tilt stimuli that presumably stimulate otolith and semicircular canals.

### Signal analysis

**Figure 3:**
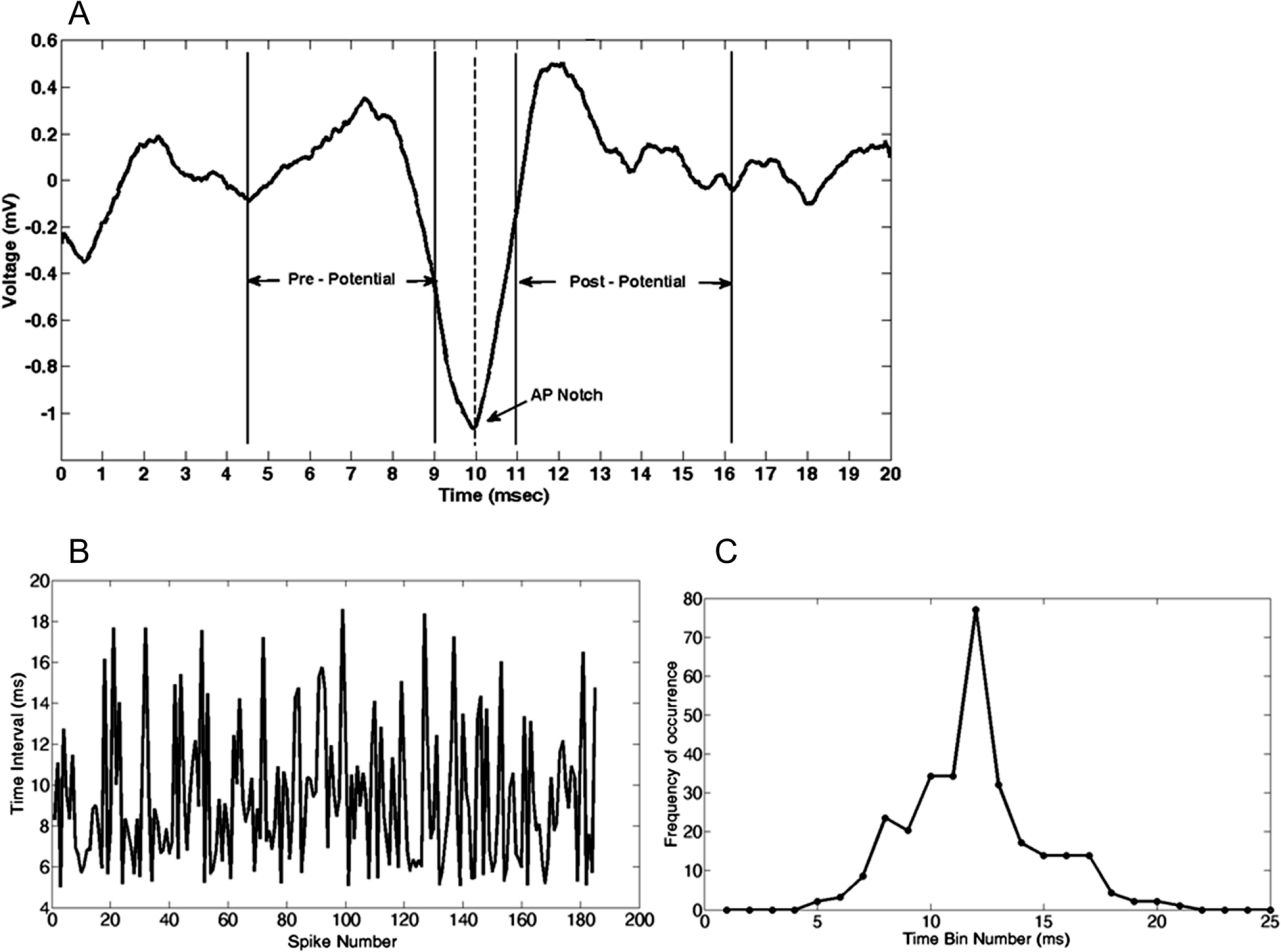
**An EVestG FP (A) and its firing pattern signals (B and C) of OnBB segment for a CTL tilt of a typical control subject. A**: The waveform's minimum point is called the action potential (AP) notch at time=10 msec. The time durations of 4.5 ms (4.5 – 9.0 ms) and 5.2 ms (11.0 – 16.2 ms) before and after the AP are considered the pre- and post- potential intervals respectively. This field potential fires repeatedly during EVestG testing and is modulated by vestibular input*.* **B**: The time interval signal of the FP occurences. **C**: The histogram of the time interval signal.

An average field potential is illustrated in Figure 3 top. Each FP fires many times representing its firing pattern. The firing pattern of the FP is presented by 1) the time intervals between each two successive FP occurrences, as in Figure 3 lower left, and 2) the probability distribution function (pdf) of the time intervals estimated by the histogram of time interval data as shown in Figure 3 lower right.

We investigated the changes in the differences among different time segments for each tilt signal to examine the effects of dynamic changes from resting to acceleration or deceleration phases of the time segment, and also the differences between the two phases (acceleration/deceleration) of chair movement as well as differences between the right and left symmetry (L-R).

The NEER algorithm [5] removes segments of the original signal that are corrupted by large artifact (due to hydraulic chair, muscle artifact, movements, poor electrode contact, etc.); therefore, not all the segments were precisely of 1.5 s duration. We excluded the segments shorter than 1.36 s. Thus, it was possible that not every subject have all the extracted features.

### Feature extraction

We calculated the mean, mean of the absolute value, variance (Var), skewness, kurtosis, HFD, entropy-based dimensions such as the Information dimension (DI) and the Correlation dimension (DC), the total energy and the average power of the aforementioned intervals for the range of 100–11000 Hz of the pre- and post-potential regions of every FP signal. Also the depth of the AP point was selected as suggested in [6].

From the time interval of the FP’s firing pattern signals (Figure 3 lower left), we calculated the mean, standard deviation (Std), skewness, kurtosis, mode, median, the DI, DC, HFD. We also calculated the average number of the time intervals less than 0.5 msec, and the correlation of the probability distribution function (PDF) of the time interval signals (Figure 3 lower right) with the relevant FP signals (Figure 3 top) as another feature.

Overall, we calculated over 40 features to consider. The features are grouped in three categories based on which signal they were calculated: 1) the features from from the field potential signals, 2) the features from one of the firing pattern representations, and 3) the features from the correlation calculation between the pdf of the time interval signals and FPs. The names of the features are summarized for the sake of space. For example, the names of “Pre Kurtosis”, “Pre mean abs”, or “Pre Energy” show that the features are found by calculation of the kurtosis, mean of absolute value, or total energy (from the entire frequency range) of the pre potential interval of the mentioned original segment.

A t-test was used to assess the statistical significance of differences for CT tilt and IT tilt between Meniere’s patients and controls. Then, we ran the mRMR algorithm on these 39 statistically significant features, and selected 5 top features (from every tilt) as the best features for classification.

### Classification (Average Voting Classifier)

Each selected feature was used in a single feature classifier using linear discriminant classification algorithm (LDA) [18]. We used leave-one-out routine [18] for training classifiers. Then, we considered each feature as a symptom, and used a heuristic method for a final classification, called Average Voting Classifier, in which every feature has “a vote” for the test subject as either Meniere’s (vote = 1) or non-Meniere’s (vote = 0) based on the LDA classifier, and the final classification is based on the average vote of all the selected best features. In this way, the final vote represents the probability that the test subject is a Meniere’s or non-Meniere’s patient. If that probability is greater than 0.5, the subject is classified as a Meniere’s patient; otherwise as non-Meniere’s.

## Results

**Figure 4:**
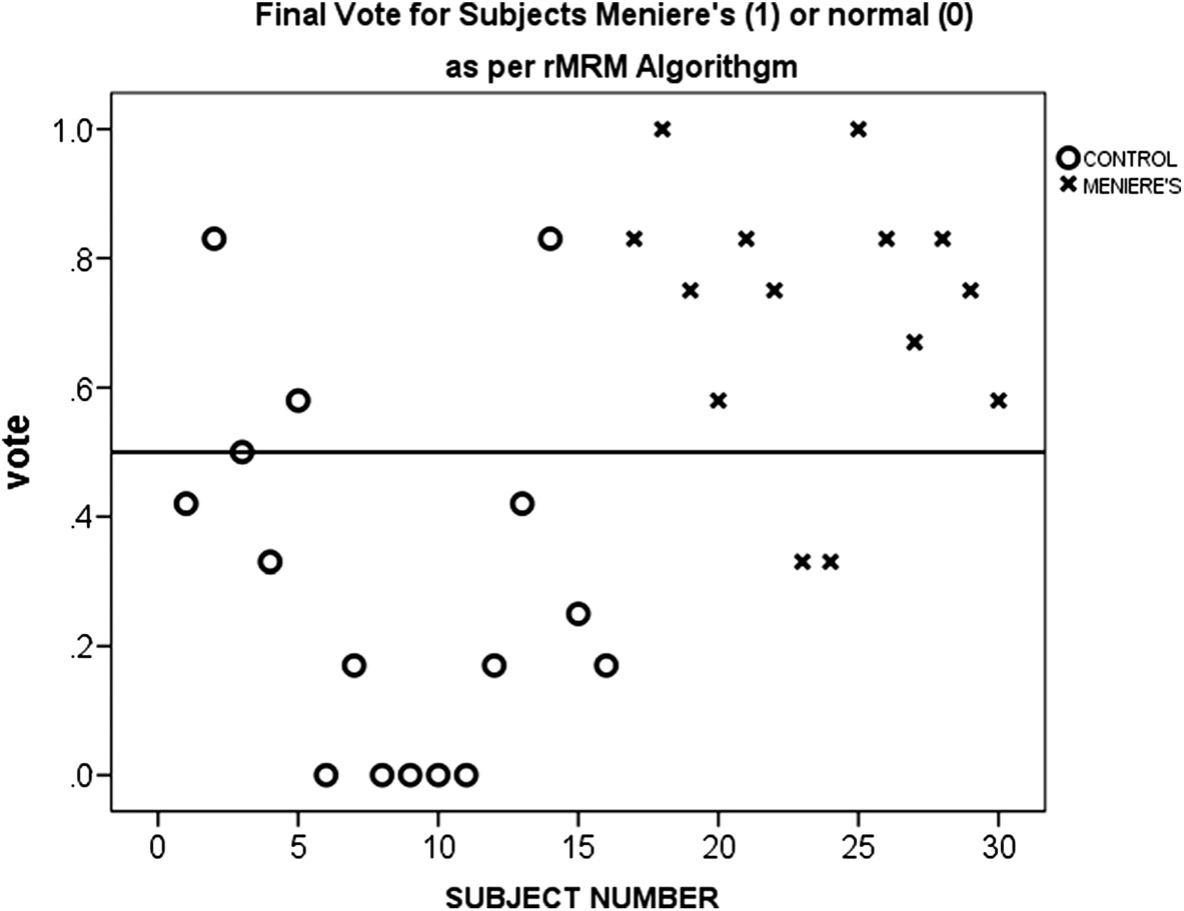
**Final Vote classification results of the training subjects for side (CT&IT) tilt for 30 subjects (14 Meniere’s patients and 16 normals).** If the probability is greater than 0.5 (above the reference line in the figure) the subject would be classified as a Meniere’s patient, otherwise the subject would be classified as normal. Sensitvity, specificity and accuracy were 85.7%, 75% and 80% respectively.

Of the features extracted from the side tilt signals, 39 (22 from CT tilt and 17 from IT tilt) were found significantly different among the Meniere’s patients and controls (*t-*test, p < 0.05). The proposed Average Vote Classifier resulted in 85.7%, 75% and 80% sensitivity, specificity and accuracy, respectively assuming the clinical diagnosis as the “gold standard” (Figure 4), which are encouraging in this first attempt. The five best features for IT and CT tilts identified by the mRMR algorithm and used for classification, are shown in Table 2.

**Table 2:** **Five best features for CT (Feature 1–5), and IT (Feature 6–10) tilts**

**Feature Number**	**Feature Name**	**Original Signal**	**p_value**
1	Post skewness	OnBB - R	0.007
2	Ap height	RTC OnAA - RTC OnBB - L-R	0.028
3	ID of Time Interval Signal	OnBB – L + R	0.007
4	Correlation	OnBB - L-R	0.048
5	Correlation	RTC OnBB - R + L	0.0079
6	Pre energy	RTC BGi - L	0.0046
7	Post HFD	RTC OnAA - R	0.0066
8	Post mean	BGi - OnBB - R	0.006
9	CD of Time Interval Signal	BGi - OnBB - R	0.0071
10	Correlation	RTC OnBB - L	0.017

The features that extracted in this study represented some consistent variations. Fractal dimension calculation (DI, and CI) over both firing patterns and FP signals showed higher values for control subjects compared to those of the Meniere’s subjects. This may imply the higher complexity in control subjects’ data compared to Meniere’s patient, which is congruent with the observed pattern of fractal dimension features calculated from other biological signals [19].

Moreover, the correlation between the FP and the pdf of FP’s firing signal demonstrated positive values in Meniere’s patients, while negative ones were observed in controls. Also, the AP was lower (wider FPs) in Meniere’s patients compared to Control ones. This may talk about possible slower conductivity of the stimulus in the vestibular organ of Meniere’s patients.

## Discussion

Tilts cause angular (rotational) accelerations and changes in the direction of gravity. For these reasons EVestG testing involves a combination of head movement responses that may or may not be consistent among patients with Meniere’s disease. Our long-term goal is to develop an objective test that is diagnostic and specific for Meniere’s disease. Our current tests, electronystagmography, rotary chair, and others, are supportive or helpful, but have no features that are unique to Meniere’s disease. We observed a distinct difference in the pre- and post-potential parts within the period time of the interest of the average FP curve of the Meniere’s and control subjects. Our finding that the fractal dimensions showed more complexity in Meniere’s patients than controls is consistent with the general physiologic literature regarding chaos theory in other organ systems – that abnormal systems lose their variability [20,21].

The results of this study show a new potential of EVestG signals toward generating an adequate set of bio-features as a diagnostic and monitoring aid for dizziness related diseases, especially Meniere’s disease. We suspect, but cannot prove at this point, that our data identify features unique to Meniere’s disease as opposed to some general findings of reduced vestibular function. If EVestG turns out to be a general method of quantifying vestibular function, it should be clinically useful. This is an ongoing study, and we hope to confirm these results with other populations. The results may lead to a simple, objective and non-invasive clinical assessment of Meniere’s disease. We acknowledge that this small dataset is not adequate to recommend clinical use without further development. The method must be tested in larger populations in future studies, which is currently under investigation at the EVestG lab at Winnipeg, Canada.

## Conclusion

Modern signal processing techniques such as EVestG may identify neural firing patterns that are diagnostic in patients with vestibular disorders but much more work needs to be done.

## Additional file

Additional file 1:
**Appendix.**
