# Supplementary material for: A new diagnostic vestibular evoked response
Source: J Otolaryngol Head Neck Surg. 2015 Apr 11;44(1):14. doi: 10.1186/s40463-015-0065-7 (PMC4403839; doi:10.1186/s40463-015-0065-7)
Supplement: Additional file 1: — Appendix. [file 40463_2015_65_MOESM1_ESM.doc]

**APPENDIX**

This appendix is intended to provide further details on some of the concepts used in the paper. The concepts have been established in the engineering or statistical or other literature and may not be familiar to otolaryngologists.

**Fractal dimension calculation:** The term “fractal dimension (FD)” can be interpreted as the “degree of meandering” (roughness, brokenness, irregularity or singularity) of an object. This object can be a geometrical figure, a process or a set of data like a time series, etc. Another interpretation of a fractal dimension is that it is the critical exponent in a power-law relation . Fractal dimension mathematically refers to a non-integer or fractional dimension of a self-similar (or a self-affine) object. The self-similarity (or self-affinity) of the object is confirmed if a portion of the object is exactly (or statistically) a scaled down version of itself.

Fractal Dimension (FD) analysis is widely used as an analytical tool in a variety of research areas particularly signal processing (of biological signals) . It measures the irregularity, the complexity and the self-similarity of the signal. As the dimensional of a line or segment is equal to one and the dimensional of a plan is equal to two, then depending on the degree of irregularity of its form, the FD of a signal (which is neither a simple line nor a plane) is a continuous real value between one and two. The more complex the signal, the higher the FD value will be.

In our work, we used Higuchi fractal dimension and Entropy based fractal dimension, which are two effective methods for calculation of FD.

**Higuchi Fractal Dimension:** Among the various fractal dimension methods, Higuchi fractal method is well suited for studying signal fluctuation in one dimension . Higuchi proposed in 1988 an efficient algorithm for measuring the FD of discrete time sequences . Higuchi’s algorithm calculates the FD directly from time series. FD can be used to quantify the complexity and self-similarity of a signal. HFD has already been used to analyze the complexity of biological signals .

Given a one dimensional time series
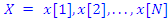
, the algorithm to compute the HFD can be described as follows:

Form
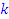
 new difference time series
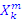
 with different lags which is defined by


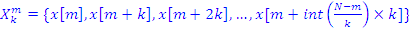
 (1)

where
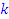
 and
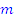
 are integers, and
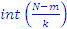
 means the integer part of the number,
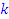
 indicates the discrete time interval between points, whereas
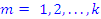
 represents the initial time value.

The length of each new time series can be defined as follows:


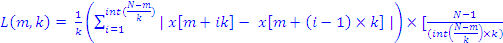
 (2)

Where
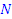
 is length of the original time series
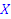
 and
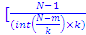
] is a normalization factor as the number of terms in a k-series varies and normalization must be used.

Then, the length of the curve for the time interval
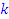
 is defined as the average of the
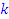
 values
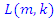
, for
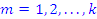
:


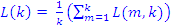
 (3)

Finally, when
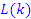
 is plotted against
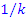
 on a double logarithmic scale, with
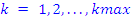
, the data should fall on a straight line, with a slope equal to the FD of
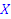
. Thus, HFD is defined as the slope of the line that fits the pairs
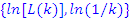
 in a least-squares sense. In order to choose an appropriate value of the parameter
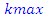
, HFD values were plotted against a range of
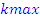
. The point at which the FD plateaus is considered a saturation point and that
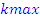
 value should be selected. A value of
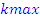
 = 8 was chosen for our study (the minimum value of
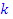
starts from 2).

**Entropy-Based Fractal Dimensions:** Entropy can be defined as the amount of information needed to specify the state of a system to a resolution of r. Entropy is known as the measure of disorder in physical systems, or an amount of information that may be gained by observations of disordered systems . Entropy-based fractal dimensions differ significantly from the morphological dimensions since they can deal with non-uniform distributions in the fractals, while the morphological dimensions show the shape of a projection of the fractal only. This is understandable because the morphological dimensions are purely metric and not probabilistic or possibilistic concepts. The information dimension and correlation dimension are special cases related to generalized entropy concept as introduced by Alfred Renyi in 1955 . Both dimensions are improvements of the geometric definition of covering a fractal object by
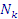
 volume elements (vels) with a diameter or radius
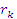
(which
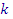
shows the order of covering).

**Information Dimension (DI):** The simplest entropy-based fractal dimension is related to the first-order Shannon entropy . Let us consider an arbitrary fractal that is covered by
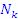
vels, each with a diameter
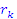
at the
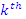
 covering. The estimation of the information dimension, *DI*, considers the density of the fractal, as determined from the relative frequency of occurrence of the fractal in each intersecting vel. If
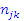
is the frequency with which the fractal enters (intersects) the
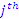
 vel of size
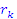
in the
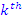
covering, then its ratio to the total number
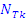
of intersects of the fractal with all the vels is an estimate of the probability
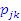
of the fractal within the
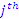
 vel, and is given by:


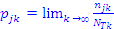
 (4)

where


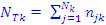
 (5)

With this probability (counted through the box-counting method ), distribution at the *k*th covering, the average (expected) self-information (i.e.,
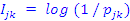
 of the fractal contained in the
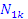
vels can be expressed by the Shannon entropy
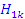
as given by:


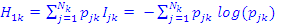
 (6)

Notice that the subscript
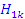
denotes that the Shannon entropy is of the first order which assumes independence between all the vels. If the following power-law relationship holds:


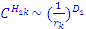
 (7)

where
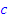
is a constant, then the information fractal dimension is


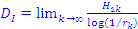
 (7a)

or


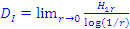
 (7b)


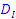
 can be obtained from the slope of a log-log plot of Shannon’s entropy
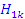
versus precision (
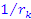
).

**Correlation Dimension (DC):** The information dimension reveals the expected spread in the non-uniform probability distribution of the fractal, but not its correlation. The correlation fractal dimension was introduced to address this problem. Let us consider a setting identical to that required to define the information dimension, *DI*. If we assume the following power-law relationship:


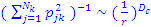
 (8)

then the correlation dimension is


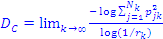
 (8a)

or


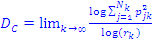
 (8b)


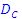
can be obtained from the slope of a log-log plot of the second-order
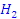
entropy versus precision
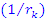
 as *DC* = *m*. It is clear that the numerator is different from the Shannon first-order entropy in the information dimension. It can be shown that it has the meaning of a correlation between pairs of neighboring points on the fractal. This correlation can be expressed in terms of a density-density correlation (or pair correlation) function. It is also known as the correlation sum, or correlation integral. This interpretation can lead to a very fast algorithm for computing the correlation dimension . There are numerous examples in the literature of computing the correlation dimension for natural fractals. Correlation dimension is, in fact, the information dimension between the pairs of points which the distance of each pair of points is less than a resolution (the diameter of a vel in a k^th^ covering). Both DI and DC represent a weighted average measure of the actual distribution of self-information over the freactal in each cover.

**Analysis of variance:** We applied statistical Analysis of Variance (ANOVA) on a randomly selected 70% subset of the data for every feature. In all statistical tests a *p-*value < 0.05 was considered significant.

**Feature selection:** We used minimal-redundancy-maximal-relevance (mRMR) feature selection method in order to reduce the number of features by investigating the best set of features among the significant ones selected from the three tilting (CT and IT) stimuli. Therefore, instead of using all available features we selectively chose a subset of features. The main advantages for feature selections can be summarized as: 1) dimension reduction (resulted in reducing the computational cost); (2) reduction of noises to improve the classification accuracy; and (3) retaining more interpretable features or characteristics that can help identify and monitor the target diseases.

There are two general approaches to feature selection: filters and wrappers . Filter type methods are essentially data pre-processing or data filtering methods. Features are selected based on the intrinsic characteristics, which determine their relevance or discriminant powers with regard to the targeted classes. Simple methods based on mutual information , statistical tests (t-test, F-test) have been shown to be effective . They also have the virtue of being easily and very efficiently computed. In filters, the characteristics in the feature selection are uncorrelated to that of the learning methods; therefore, they have better generalization property.

In wrapper type methods, feature selection is "wrapped" around a learning method: the usefulness of a feature is directly judged by the estimated accuracy of the learning method. One can often obtain a set with a very small number of non-redundant features , which gives high accuracy, because the characteristics of the features match well with the characteristics of the learning method. Wrapper methods typically require extensive computation to search the best features.

The heuristic mRMR method is proved to achieve the two mentioned goals, a set of efficient (obtaining high classification accuracy) features which have the least redundancy, simultaneously through selecting (adding) one feature at a time called “first-order” incremental search . In this method a set *S* of *m* features
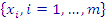
 is identified by jointly selecting the features with the highest relevance to the target class *c* (obtaining minimal classification error or maximal accuracy) and with the minimal redundancy (obtaining almost uncorrelated features which are maximally representative of the original space covered by the entire dataset). The maximum relevance and the minimum redundancy criteria are both measured based on mutual information calculation .

In case of two random variables *x* and *y*,
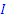
 is defined in terms of their probabilistic density functions
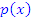
,
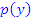
, and
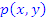
as follows:


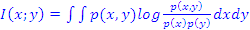
 (9)

Maximum relevance (
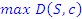
) criterion is defined by the mean value of all mutual information values between individual feature
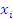
and class *c* as follows:


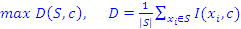
 (10)

It is likely that features selected according to Max-Relevance could have rich redundancy, i.e., the dependency among these features could be large. Therefore, the following minimal redundancy (
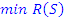
) criterion can be added to select mutually exclusive features .


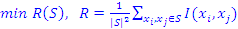
 (11)

In this approach, the first feature is selected due to the Max-Relevance criterion. Then, an iterative search method adds one feature at a time such that by having a set of *m-1* feature space, *S_m-1_*, the *m^th^* feature is chosen to achieve the two equations 10 and 11. The criterion which is combining the above two constraints is called Min-Redundancy-Max-Relevancy (mRMR) , and simultaneously optimizes D and R spaces as below:


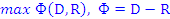
 (12)

Using mRMR method we selected the first 5 features in the sorted list of features from every tilt. This gave us 5 best features of contralateral, ipsilateral and back tilts separately.Using mRMR method we selected the first 5 features in the sorted list of features from every tilt. This gave us 5 best features of contralateral, ipsilateral and back tilts separately.
